# Modulation of Radiation Injury Response in Retinal Endothelial Cells by Quinic Acid Derivative KZ-41 Involves p38 MAPK

**DOI:** 10.1371/journal.pone.0100210

**Published:** 2014-06-23

**Authors:** Jordan J. Toutounchian, Jena J. Steinle, Patrudu S. Makena, Christopher M. Waters, Matthew W. Wilson, Barrett G. Haik, Duane D. Miller, Charles R. Yates

**Affiliations:** 1 Department of Pharmaceutical Sciences, University of Tennessee Health Science Center, Memphis, Tennessee, United States of America; 2 Department of Ophthalmology, University of Tennessee Health Science Center, Memphis, Tennessee, United States of America; 3 Department of Anatomy and Neurobiology, University of Tennessee Health Science Center, Memphis, Tennessee, United States of America; 4 Department of Physiology, University of Tennessee Health Science Center, Memphis, Tennessee, United States of America; UAE University, Faculty of Medicine & Health Sciences, United Arab Emirates

## Abstract

Radiation-induced damage to the retina triggers leukostasis, retinal endothelial cell (REC) death, and subsequent hypoxia. Resultant ischemia leads to visual loss and compensatory retinal neovascularization (RNV). Using human RECs, we demonstrated that radiation induced leukocyte adhesion through mechanisms involving p38MAPK, p53, and ICAM-1 activation. Additional phenotypic changes included p38MAPK-dependent tyrosine phosphorylation of the focal adhesion scaffolding protein, paxillin (Tyr118). The quinic acid derivative KZ-41 lessened leukocyte adhesion and paxillin-dependent proliferation via inhibition of p38MAPK-p53-ICAM-1 signaling. Using the murine oxygen-induced retinopathy (OIR) model, we examined the effect of KZ-41 on pathologic RNV. Daily ocular application of a KZ-41-loaded nanoemulsion significantly reduced both the avascular and neovascular areas in harvested retinal flat mounts when compared to the contralateral eye receiving vehicle alone. Our data highlight the potential benefit of KZ-41 in reducing both the retinal ischemia and neovascularization provoked by genotoxic insults. Further research into how quinic acid derivatives target and mitigate inflammation is needed to fully appreciate their therapeutic potential for the treatment of inflammatory retinal vasculopathies.

## Introduction

Radiation retinopathy (RR) is a chronic degenerative disease that leads to significant visual impairment [1], [2]. RR results from exposure of the eye to various directed radiotherapy interventions such as external beam, plaque brachytherapy, and gamma knife [3]–[5]. Radiotherapy is used to treat uveal melanoma since it provides both equivalent local tumor control and survival enucleation (eye removal) [6], [7]. The incidence of RR in patients with uveal melanoma treated with plaque brachytherapy has been estimated at 20% with a subset of these patients developing proliferative neovascularization [8]–[10]. RR results in treatment related visual loss and in cases of severe neovascularization can cause glaucoma, necessitating secondary enucleation for a blind, painful eye [11].

**Figure 1:**
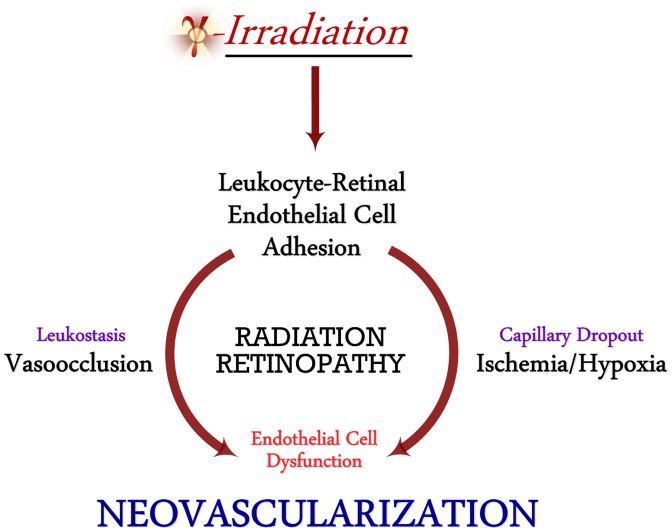
Pathophysiology of Radiation Retinopathy. Radiation to the eye triggers leukocyte adhesion, blockage, and nutrient/oxygen deprivation of retinal vasculature. Resultant hypoxia leads to dysfunctional retinal neovascularization and vision loss.

Radiation-induced damage to the vascularized retina triggers an exuberant pro-inflammatory response resulting in leukocyte adhesion and stasis, vessel occlusion, retinal endothelial cell (REC) death, and subsequent hypoxia [12], [13]. Clinical features of a progessive ischemic retinopathy include vascular leakage and capillary non-perfusion, attributed in large part to the accumulation of immune cells in the damaged areas [14]. The ischemic retina can then trigger a subsequent growth-factor mediated neovascularization. Studies inhibiting adhesive interactions using antibodies against ICAM-1 prevented retinal endothelial cell dysfunction, death and subsequent tissue ischemia, in turn, preventing compensatory retinal neovascularization (Fig. 1) [15].

Gamma(γ) radiation-induced DNA double strand breaks (DSBs) trigger phosphorylation of p38MAPK and accumulation of p53 in human endothelial cells. The p38MAPK stress kinase pathway plays an indispensable role in promoting inflammatory responses elicited by DNA damaging stressors such as chemotherapeutics, oxidative stress, and radiation [16]. Furthermore, p38MAPK-dependent phosphorylation at serine residues at the N-terminus region of p53 has been shown to enhance its stability, accumulation and activation [17]–[20]. Activated p53 binds to its cognate DNA response element and promotes the transcription of inflammatory and apoptotic genes, such as ICAM-1 [18]. Activation of this pathway has been linked to pro-apoptotic signaling and transcriptional events promoting p53-dependent cell cycle arrest, inflammation and/or cell death [21], [22]. Inhibiting p38MAPK signaling in other cell systems impairs DNA-binding and transcriptional activity of p53 leading to a reduction in both inflammatory and pro-apoptotic signaling [18], [20], [23].

We have identified a novel quinic acid derivative KZ-41 as a promising radiomitigant that provides a substantial survival benefit following total body irradiation (TBI; LD_80/30_) as well as enhanced vascular repair mechanisms in a murine combined radiation and vascular injury model [24]–[26]. In an *in vitro* model of genotoxic stress using the alkylating agent melphalan, we have shown KZ-41 to specifically counteract p38MAPK-dependent pro-apoptotic and inflammatory signaling in primary human RECs [16]. In this study, we further investigated the cell signaling mechanisms by which quinic acid derivatives modulate the radiation injury response.

## Materials and Methods

### Reagents/Antibodies

KZ-41 was synthesized in Dr. Duane Miller's laboratory and verified to be >96% pure by nuclear magnetic resonance spectroscopy [27]. Calcein-AM was obtained from BD Biosciences (San Jose, CA). Conjugated ICAM-1 (sc-107 PCPC5) antibody for confocal microscopy was purchased from Santa Cruz Biotechnology (Santa Cruz, CA). DAPI nuclear stain was obtained from Pierce (Rockford, IL). Phosphorylated (Thr180/Tyr182) p38MAPK primary antibodies were purchased from R&D Systems (Minneapolis, MN). Phosphorylated (Ser-15, -33, -37) and total p53, p38MAPK, unconjugated ICAM-1, phosphorylated (Tyr118) and total paxillin, and GAPDH primary antibodies were acquired from Cell Signaling (Danvers, MA). Alpha-tubulin primary antibody and secondary antibodies, IRDye 800CW goat anti-rabbit, and IRDye 680LT goat anti-mouse were purchased from LI-COR Biotechnology (Lincoln, NE). The non-selective p38MAPK inhibitor SB202190 was purchased from Tocris Bioscience (Bristol, UK).

### Cell-Culture

Primary human retinal microvascular endothelial cells (RECs, Lot 181) were acquired from Cell Systems Corporation (CSC, Kirkland, Washington). Cells were grown on attachment factor (AF)-coated surfaces in M131 medium containing microvascular growth supplements (MVGS), gentamicin (10 mg/mL), and amphotericin B (0.25 mg/mL) (Invitrogen; Carlsbad, CA). Only primary cells within passage six were used. U937 (human monocytic-like) cells (ATCC CRL-1593.2, Manassas, VA) were cultured in RPMI 1640 (Invitrogen) supplemented with 10% fetal bovine serum, penicillin (5000 IU) and streptomycin (5 mg/mL). U937 cells to passage 10 were used for adhesion experiments [28].

For immunoassays, RECs were plated into six-well plates and cultured for two days. RECs were pre-treated with KZ-41 (10 µM) for 12 hours and then exposed to gamma (γ) radiation (30 Gray; Gy) using a Shepherd Mark I, model 68, ^137^Cs irradiator (J.L. Shepherd and Associates, San Fernando, CA) at a dose rate of approximately 3 Gy/min. The non-selective p38MAPK inhibitor SB202190 [29] was added to culture medium 30 minutes prior to irradiation.

### Static cell adhesion

Cellular adhesion under static conditions was assessed using a microplate assay [30], [31]. Briefly, human primary RECs (10^5^ cells/well) were seeded to 96-well plates and cultured to confluence. RECs were treated with either KZ-41 (10 µM) or vehicle (0.9% normal saline), irradiated (30 Gy), and incubated for 24 hours at 37°C. Calcein-AM-loaded U937 cells were added to REC-containing wells and allowed to adhere for 30 minutes. Non-adherent cells were removed from wells by gentle washes with phosphate-buffered saline (PBS) and adhesion was quantified using a fluorescence microplate reader (excitation/emission wavelengths of 485/535 ηm). Data represent mean fluorescence ± standard deviation (SD) normalized to background fluorescence.

### Parallel-Plate Flow chamber

Cell adhesion under physiological fluid-shear was investigated using a parallel-plate flow chamber and continuous flow-loop (Cytodyne Inc., La Jolla, CA) at a shear stress of two dyne/cm^2^ [32], [33]. Shear stress within the chamber was determined using a constant fluid flow-rate calibrated by adjusting the height of the hydrostatic inlet and outlet ports of the fluid reservoir [34], [35]. The flow rate for the required shear stress was calculated using the following equation: SS = 6Q µ/bh^2^, where SS =  shear stress (dyne/cm^2^), Q =  flow rate (cm^3^/s), μ =  fluid viscosity (dyne * s/cm^2^), b =  chamber width (cm), h =  chamber height (cm). RECs were seeded onto AF-coated microscope slides (75×38 mm; Corning Inc., Corning, NY) and grown to confluence. KZ-41 (10 µM) or vehicle-treated RECs were irradiated (30 Gy) and incubated for 24 hours. Slides were then placed into the chamber and U937 cells (2.5×10^6^ cells/mL) were perfused over the REC monolayer. Interacting cells were monitored over two hours using at least six different fields of view and digitally recorded for off-line analysis. Phase contrast images of adherent cells were obtained using a Nikon Diaphot 300 phase-contrast microscope (Nikon, Melville, NY) equipped with a Dage-MTI series 68 camera (Dage-MTI, Michigan City, IN). High-resolution video and images were analyzed using Adobe Premier Pro CS5.5 (Adobe Systems; San Jose, CA). Firm adhesion was defined as interacting cells remaining stationary at the end of two hours, then counted and averaged over at least six fields of view [36], [37]. After two hours, RECs were removed from the flow chamber and fixed in 4% paraformaldehyde for 15 minutes at room temperature and washed three times with ice-cold PBS. Data from three separate experiments represent mean adherent cells/fields of view ± SD.

### Immunofluorescence and Confocal microscopy

Non-specific blocking of proteins on cellular surface was done using 10% bovine serum albumin (BSA) containing blocking buffer for at least one hour at room temperature. Human anti-ICAM-1 antibody conjugated to PerCp-Cy5.5 was diluted in PBS (1∶50) and incubated with the slide for one hour at room temperature with gentle rocking. Slides were then washed twice with cold PBS and incubated with DAPI nuclear stain for 10 minutes. Cells were again washed and mounting medium along with cover slips were added to slides and sealed prior to imaging. A Zeiss LSM 710 system with Zen 2010 v.6.0 software (Carl Zeiss Microscopy; Thornwood, NY) was used in image acquisition and analysis.

### Immunoblot (Western blot) analysis

Irradiated RECs with or without treatments of either KZ-41 and/or SB202190 were carried out at 30 Gy. For ICAM-1 protein level analysis, REC lysates were collected 24 hours after IR. For phosphorylation status of p38MAPK and p53 stress pathways, REC lysates were collected four hours following exposure to IR. Unirradiated RECs were taken out of the incubator during irradiations for environmental controls. Cellular proteins were analyzed by Western blot after SDS-PAGE using human specific primary antibodies. REC lysates were collected in 1X RIPA lysis buffer (50 mM Tris·HCl, pH 7.4, 150 mM NaCl, 2 mM EDTA, 1% Nonidet P-40, 0.1% SDS) with protease/phosphatase inhibitor (1X) cocktail (Roche; Indianapolis, IN). Lysates were kept on ice and centrifuged at 10,000 g for 10 minutes and cell free lysates were kept at −80°C until further analysis. Total protein concentration was measured by BCA assay (Pierce, Rockford, IL). Protein samples were mixed with 4X LDS loading buffer with 2.5% 2-mercaptoethanol (Sigma), heated to 70°C for 10 minutes, and loaded on a NuPAGE 4–12% Bis-Tris gel (Invitrogen). Immunoblotting was performed with nitrocellulose membranes (Bio-Rad) at 170-mA start and 110-mA end at 25 V for two hours in NuPAGE transfer buffer (Invitrogen) containing 20% methanol. Membranes were blocked using Odyssey blocking buffer (LI-COR) for one hour at room temperature with gentle rocking. Membranes were then incubated at 4°C with specific primary antibodies (1∶1000) overnight. Cellular protein was normalized using GAPDH (Cell Signaling) or α-Tubulin (LI-COR) [1∶20,000]. Secondary antibodies (IRDye 800CW goat anti-rabbit and IRDye 680LT goat anti-mouse) (LI-COR) [1∶10,000] were incubated in the dark at room temperature for 45 minutes. Dual-channel infrared scan and quantitation of immunoblots were conducted using the Odyssey Sa infrared imaging system with Image Studio (Ver. 3.1.4) (LI-COR).

### REC proliferation Assays

To evaluate KZ-41 modulation of irradiation-induced retinal endothelial cell proliferation, 50,000 cells with or without KZ-41 (10 µM) were plated into each well of a 96-well dish, irradiated at 30 Gy and incubated for 24 hours. Following treatment with KZ-41 or vehicle, cellular proliferation was determined using the tetrazolium salt WST-1 and a microplate reader (UQuant Reader; BioTek, Winooski, VT) according to the assay manufacturer's instructions (Cell Proliferation Assay Kit, WST dye, ELISA based; Millipore, Billierca, MA) at 450 ηm. The absorbance at 450 ηm (recorded as mean OD ± SD) is directly correlated with cellular proliferative capacity.

### Ethics Statement

All animal experimentation was performed under the guidelines of the Association for Research in Vision and Ophthalmology for the humane use of animals in vision research. The study was approved by the UTHSC Institutional Animal Care and Use Committee (IACUC) in accordance with established guidelines. Eye enucleation was performed under isoflurane anesthesia and all efforts were made to minimize suffering.

### Murine oxygen-induced retinopathy (OIR) model

C57BL/6J (The Jackson Laboratory, Bar Harbor, ME) mouse pups used in these experiments were housed with nursing mothers for the entire study period and given food and water *ad libitum*. Ocular nanoemulsion (NE) used for drug delivery comprised Capryol 90 (7.5% v/v), Triacetin (7.5% v/v), Tween-20 (17.5% v/v) and Transcutol P (17.5% v/v) (Gattefossé Pharmaceuticals, Saint-Priest, France) generated via homogenization and water titration methods, as previously described [38], [39].

Retinal neovascularization (RNV) was induced using a mouse model of oxygen-induced retinopathy (OIR) [40], [41]. Mouse pups were randomly divided into four separate groups: 1) Untreated mice under ambient normal oxygen (normoxia) conditions (negative-control); 2) Untreated mice exposed to hyperoxia conditions (positive-control); 3) Nanoemulsion vehicle treated hyperoxia-exposed mice (vehicle-control); and 4) Hyperoxia-exposed mice treated with KZ-41-loaded nanoemulsion (compound-treated). A minimum of five animals were used for each experimental group. C57BL6/J mouse pups were placed in a Plexiglas chamber and exposed to 75% oxygen maintained and automated by an oxygen controller (Pro-Ox, model P110; Biospherix, Lacona, NY) at post-natal day seven (P7) for five days and then returned to normal oxygen (P12). OIR mice received daily ocular administration of either KZ-41 (100 mg/kg)-loaded nanoemulsion, vehicle (ocular nanoemulsion) or left untreated from P12 to P17. Normoxia (negative controls) mice were not manipulated during the study period. On P17, mice were anesthetized under isoflurane and both eyes were removed, followed by sacrifice. Retinas were harvested, mounted, and stained to investigate retinal angiogenesis [42], [43]. For retinal whole mounts, enucleated eyes underwent weak fixation (for ease of hyaloid vasculature removal) in 4% paraformaldehyde (PFA) in PBS for one hour on ice and washed three times. Retinas were then isolated and mounted onto microscope slides. Whole retinas were incubated overnight at 4°C with isolectin B4-594 (Alexa Fluor 594; Molecular Probes, Eugene, OR). Isolectin-stained retinas were then washed three times in 1X PBS, sealed on slides using Prolong Gold (Invitrogen), and imaged.

Images were acquired using a Nikon Eclipse 80i confocal microscope and analyzed with Nikon-NIS elements software (Nikon) [44]. Quantification of avascular area (AV) and neovascularization (NV) in retinal whole mounts was performed in Adobe Photoshop (Adobe Systems, Inc.) [42], [43]. Briefly, the AV area was determined by the absence of isolectin staining surrounding the optic disc. The area devoid of vascularization was characterized as a percentage of total retinal area (%AV). Quantification of NV was determined after threshold limits were set within software parameters. This technique ensured the quantification of only clusters and tufts of NV while excluding the normal vascularized retina (less intense staining). Photoshop analysis tools were used to manually outline NV formations and data was recorded as a percentage of total retinal area (%NV) [42], [43].

### Statistical analyses

All data represented herein were performed in replicates of three and presented as the mean ± standard deviation (SD), unless otherwise indicated. Analysis of variance (ANOVA) with Scheffe's post-hoc test was used to compare mean values. Statistical significance was set at P<0.05.

## Results

### Radiation induces adhesion of U937 monocytic cells under static and dynamic flow conditions

**Figure 2:**
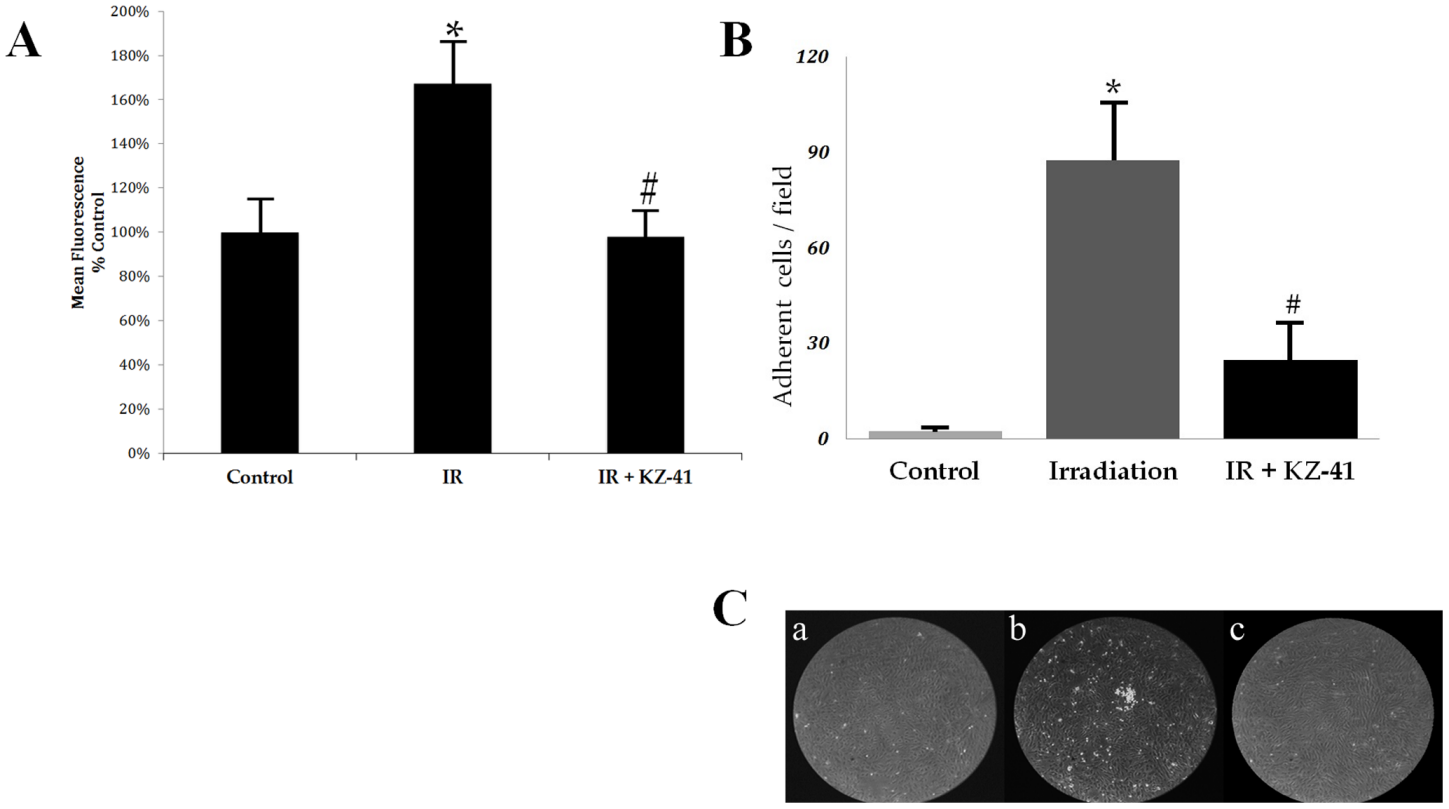
Radiation-induced REC-U937 adhesion. Panel A) Static-adhesion: Irradiated human RECs were incubated for 24 hours in culture medium containing vehicle (PBS) or KZ-41 (10 µM). Calcein-AM loaded U937 cells were co-cultured with RECs (n = 8/group) for 30 minutes and non-adherent cells were washed from wells; attached U937 cells were quantified with a fluorescence microplate reader (excitation/emission wavelengths of 485/535 ηm). Data demonstrate that KZ-41 inhibits IR-induced leukocyte attachment to RECs (*, **P<0.005) (All data normalized to background fluorescence; data represent % Control fluorescence signal ± SD). Panel B) Flow-Chamber adhesion: RECs were irradiated and cultured for 24 hours. U937 cells were perfused over RECs placed in flow-chamber and digital images were collected after two hours. Data represent mean #-adherent cells/field ± SD. KZ-41 (10 µM) treatment significantly decreased IR-induced adhesion of U937 cells (*, #P<0.05). Panel C) Representative still images A) Control, B) IR-REC and C) IR+KZ-41 show extent of U937 accumulation on surface of RECs.

A key mechanism of radiation-induced retinal injury involves leukocyte entrapment and accumulation within microvascular circulation [45], [46]. We used a fluorescence-based static-adhesion assay to determine whether or not radiation induced an adhesive phenotype in RECs (Fig. 2a). Adhesion of U937 monocytic cells was enhanced by nearly 70% in irradiated RECs (P<0.005). Addition of KZ-41 immediately prior to radiation led to a substantial decrease in adherent cells (P<0.005).

Immunoglobin superfamily of cellular adhesion molecules (*e.g.*, ICAM, VCAM, PECAM, *etc.*) and the selectin family (*i.e.*, L-, P- and E-selectin) are able to perform comparable adhesive interactions in static environments where the lack of shear stress promotes strong cell-cell contacts [46], [47]. Therefore, we used a parallel-plate flow chamber and adapted continuous flow-loop (Cytodyne) to establish a dynamic fluid environment of circulating leukocytes allowing for the observation and quantification of the three characteristic events: tethering, rolling and firm adhesion [48]. Twenty-four hours after radiation, RECs were placed in the flow chamber and interacting U937 cells were observed and quantified via phase-contrast microscopy. After two hours, digital analysis of U937 cell adhesion to the REC monolayer revealed significant increases following radiation compared to unirradiated RECs (Fig. 2b, 2±2 vs. 87±18 adhered cells; *P<0.05). Treatment with KZ-41 significantly reduced U937 adherence (Fig. 2b, 25±12 vs. 87±18 adhered cells; #P<0.05). Representative still images from flow chamber experiments are presented in Figure 2C to show extent of U937 adhesion (A-C; Control, IR, IR+KZ-41, respectively).

### Radiation enhances ICAM-1 levels in RECs

**Figure 3:**
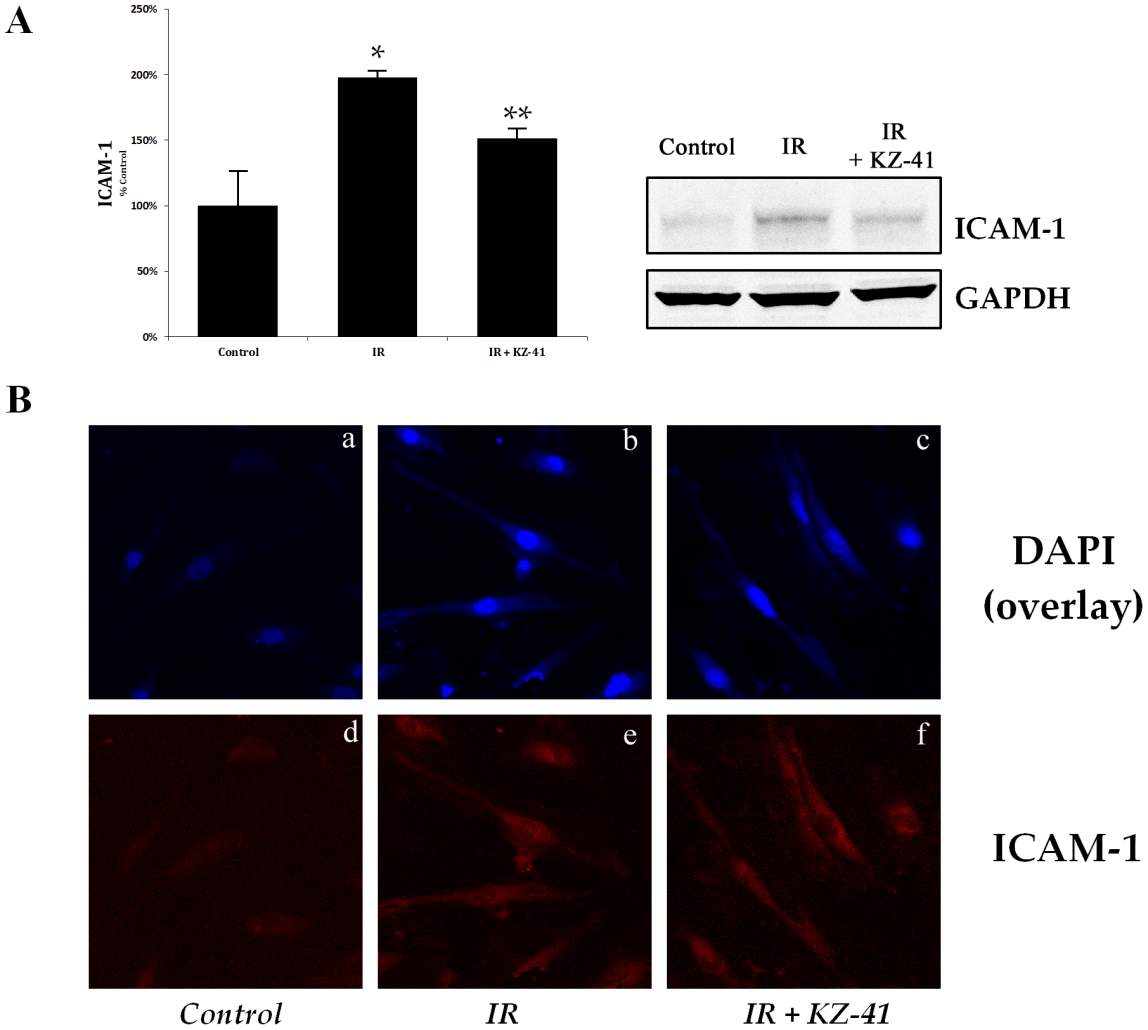
ICAM-1 up-regulation in irradiated RECs. A) Immunoblotting of ICAM-1 from IR-RECs after 24 hours show a significant up-regulation compared to unirradiated cells; treatment with KZ-41 (10 µM) reduces ICAM-1 levels by nearly 24% (Mean ± SD; *P<0.05, **P<0.05; n = 3).B) Confocal microscopy of RECs from flow-chamber slides. Top panels (A-C; Control, IR and IR+KZ-41, respectively) represent overlay images of DAPI and ICAM-1. Lower panels, D-E represents ICAM-1 immunoreactivity.

Numerous in vitro and in vivo models of radiation-induced vascular injury have established ICAM-1 regulation as an important pathological indicator of inflammation [46], [49], [50]. Therefore, we investigated ICAM-1 protein levels in RECs 24 hours post-IR. ICAM-1 levels were significantly enhanced by radiation (Fig. 3a; P<0.05 vs. unirradiated RECs) and were reduced with treatment of KZ-41 compared to irradiated RECs (P<0.05). Immunoreactivity to ICAM-1 protein was then investigated in RECs taken from flow-chamber experiments (Fig. 2). Confocal images further suggest that radiation-induced regulation of ICAM-1 is modulated by KZ-41 treatment (Fig. 3b).

### KZ-41 inhibits radiation-induced ICAM-1 expression through a p38MAPK-dependent mechanism

**Figure 4:**
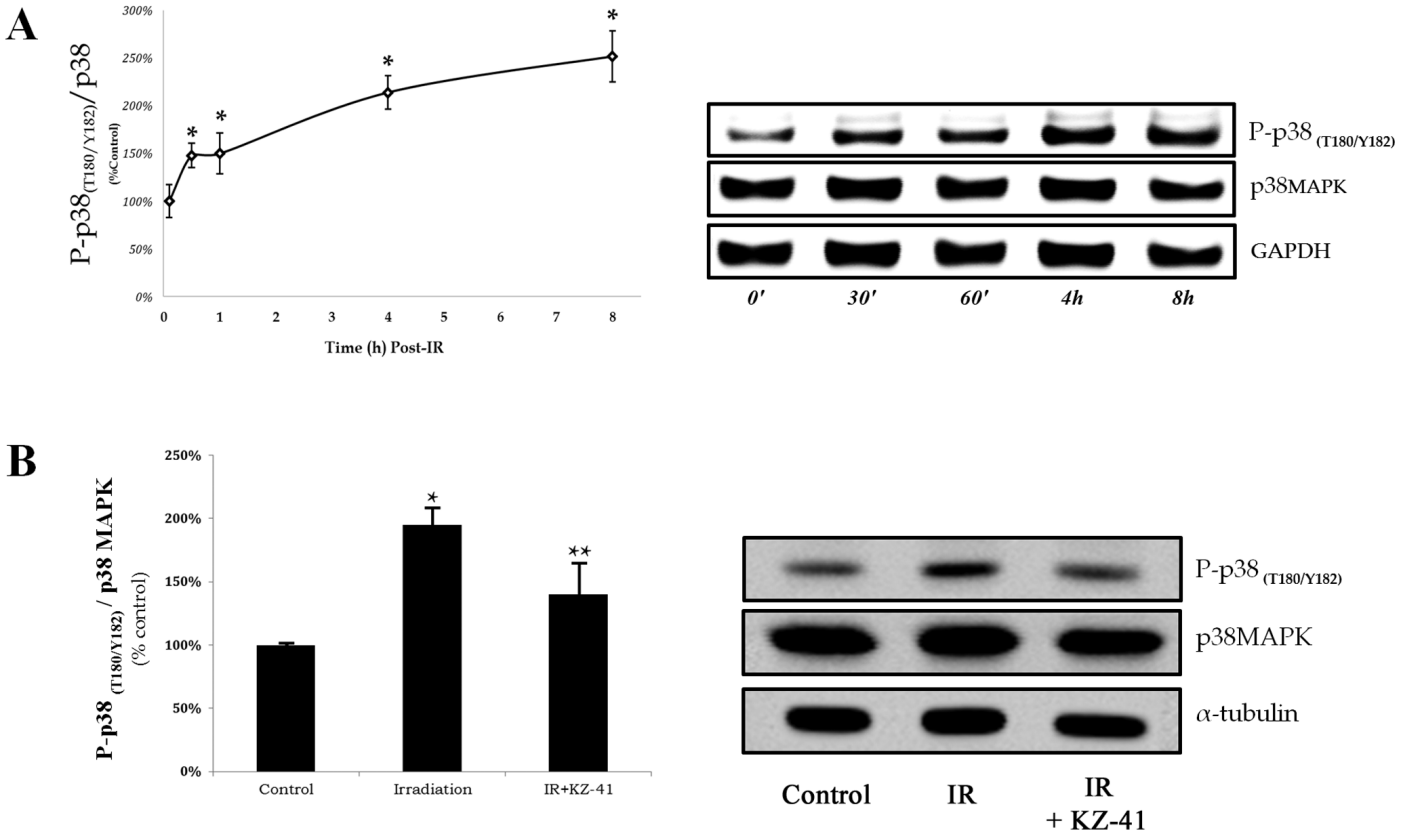
Induction of p38MAPK phosphorylation in irradiated RECs. A) RECs receiving 30 Gy irradiation in a single fraction show increases in phosphorylation of p38MAPK (T180/Y182) that reach a plateau between 4–8 hours compared to control cells at same time-points (*P<0.05). B) Irradiated RECs with or without treatment of KZ-41 (10 µM) were harvested and analyzed for phospho-p38MAPK at 4 hours. KZ-41 treated RECs showed significant reductions in total levels of phosphorylated p38MAPK (T180/Y182), as compared to IR-RECs (**P<0.05).

In response to DNA damaging events (*e.g.*, radiation), the stress kinase p38MAPK is activated by dual phosphorylation at Thr180 and Tyr182 to promote ICAM-1 protein expression [51]–[54]. Previously, we demonstrated that melphalan-induced ICAM-1 protein expression was reduced with an inhibitor specific to phosphorylated-p38MAPK (SB202190; 10 µM) [16]. Based on these data, we hypothesized that KZ-41 reduction in IR-induced ICAM-1 expression was attributed to modulation of p38MAPK-dependent inflammatory signaling. We first performed a time-course experiment to both confirm and identify maximum p38MAPK phosphorylation (Thr180/Tyr182) post-irradiation (Fig. 4). Relative to total p38MAPK levels, phosphorylated-p38MAPK reached a transient plateau over four to eight hours post-IR (Fig. 4a). We then collected and analyzed treated cells four hours following irradiation. In comparison, irradiated RECs that were treated with KZ-41 prior to exposure had 30% reduction in phosphorylated-p38MAPK (Fig. 4b; P<0.05).

### Radiation activates p53 in RECs

**Figure 5:**
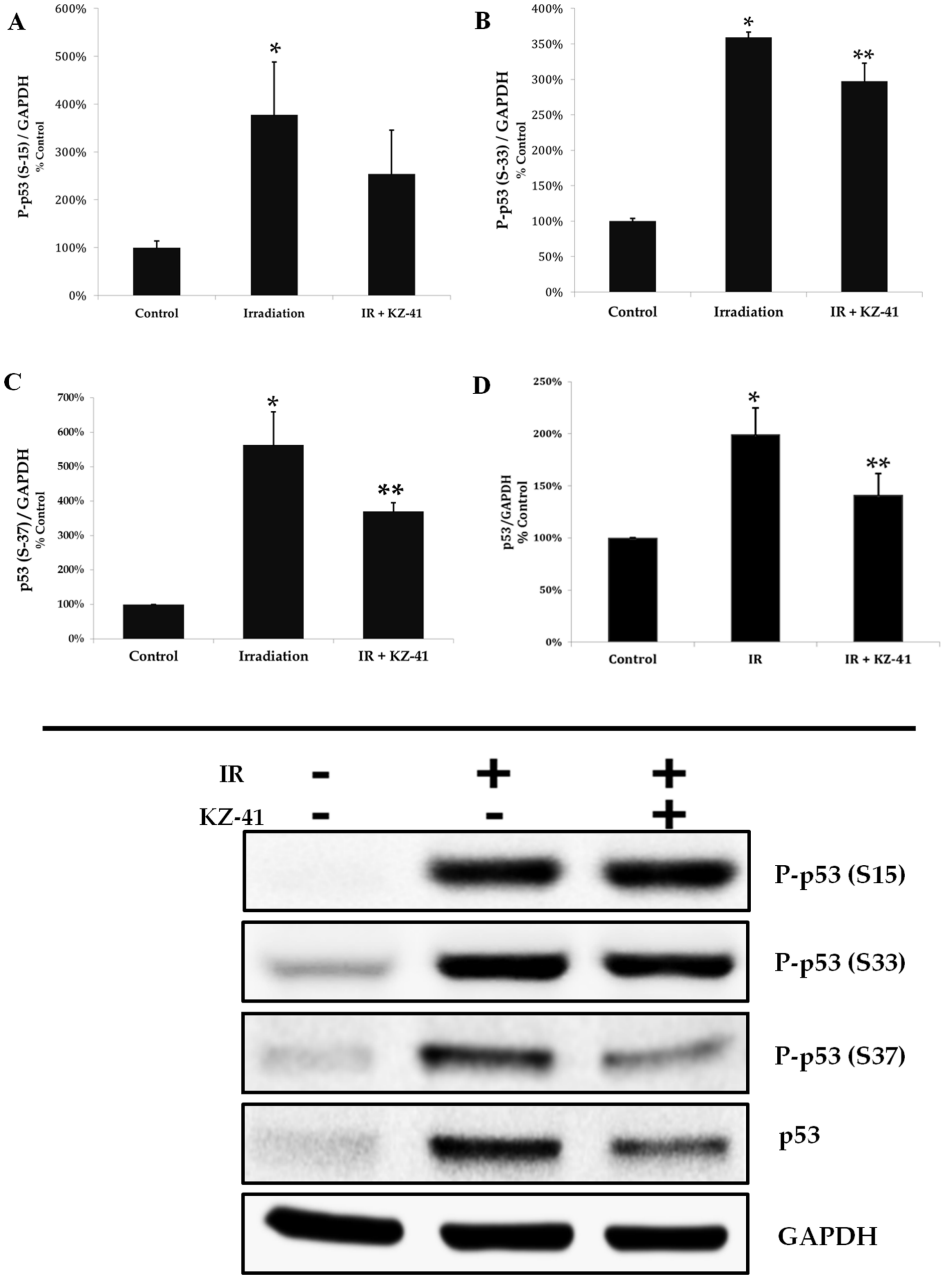
Radiation-induced p53 phosphorylation and accumulation. A–C) Phosphorylation at serine 15, 33, 37 was significantly induced by irradiation (*P<0.05) after 4 hours. The ratio of p53 phosphorylation at Ser 33 and 37 (relative to GAPDH) in KZ-41-treated (10 µM) RECs showed significant reduction (**P<0.05). D) Total p53 protein accumulation revealed significantly reduced levels in KZ-41 treated RECs (**P<0.05).

Radiation-induced phosphorylation of p53 at serine residues 15, 33, and 37 stabilizes p53 by preventing MDM2-driven polyubiquitination and degradation and has been shown to require the kinase activity of p38MAPK [17], [20]. In RECs, radiation dramatically increased p53 phosphorylation at serine residues 15, 33, and 37, which in turn, led to accumulation of total p53 (Fig. 5). The net result was an increased ratio of phosphorylated to total p53 for each of the serine residues examined. Next, we hypothesized that KZ-41, by virtue of inhibiting p38MAPK, reduces radiation-induced serine phosphorylation and accumulation of p53. As hypothesized, pre-incubation with KZ-41 (10 µM) diminished p53 phosphorylation and accumulation (Fig 5).

### Radiation induces a paxillin-dependent proliferative phenotype

**Figure 6:**
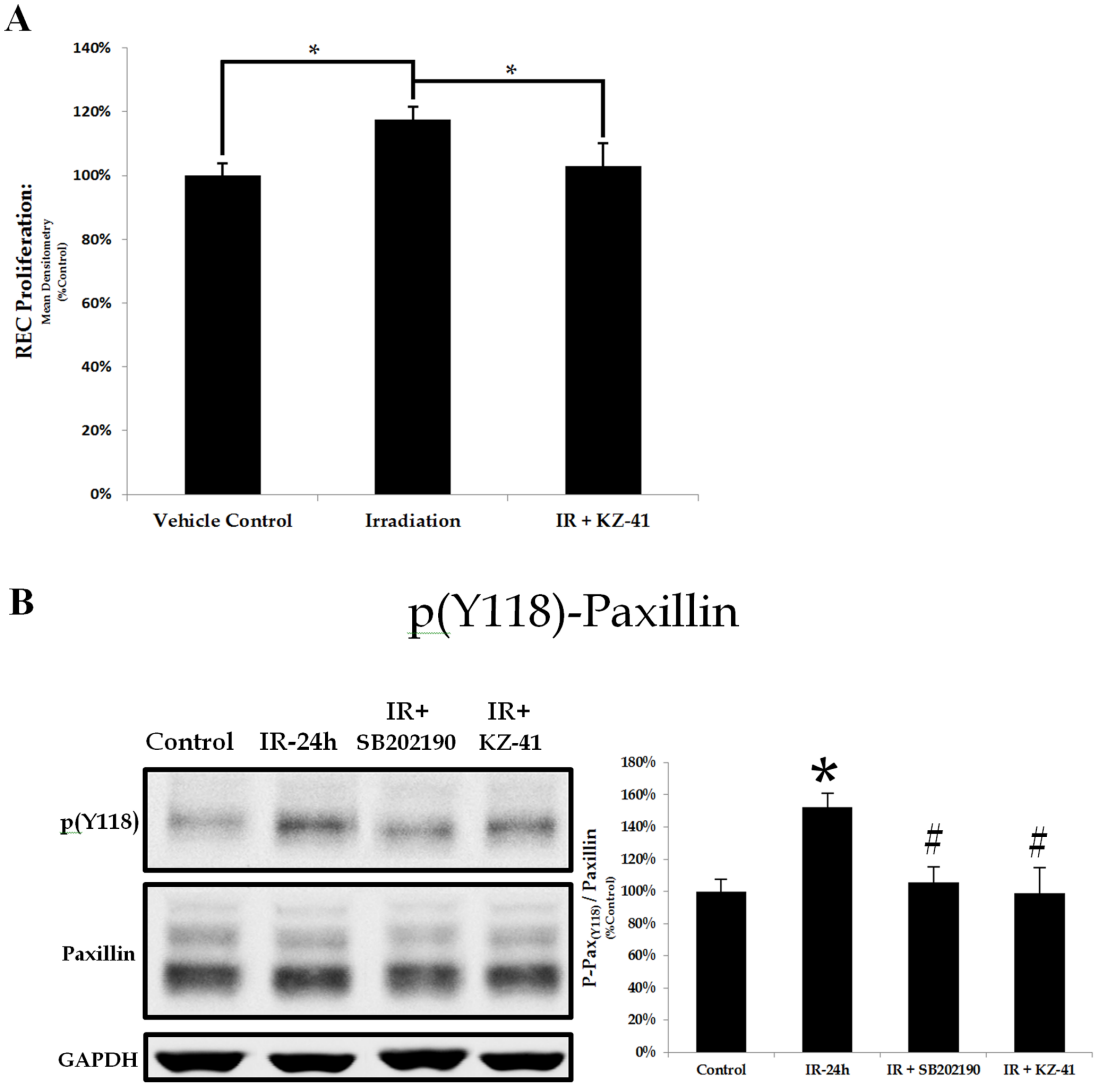
Radiation-induced paxillin-dependent proliferative capacity/phenotype. A) Irradiation-induced REC proliferation was measured after 24 hours using the WST-1 proliferation assay. REC proliferation was enhanced by irradiation and was significantly reduced with treatment of KZ-41 (10 µM) (*P<0.05). B) Paxillin phosphorylation (Y118) was measured 24 hours after irradiation using immunoblotting and showed enhanced levels. Both KZ-41 (10 µM) and p38MAPK inhibitor SB202190 (10 µM) significantly reduced levels of paxillin phosphorylation (*P<0.01, #P<0.05).

Irradiation of the retinal vasculature triggers cell death in a small fraction of cells which absorb the brunt of the ionizing radiation [55]. Similarly, we found that a minority of irradiated RECs exhibited signs of apoptosis (∼20%; <24 hours) (data not shown). Surviving RECs elicited a compensatory migratory/proliferative response, which leads to extensive vascular remodeling and pathologic neovascularization (Fig. 1) [2], [56]. Using an in vitro assay we showed radiation-induced proliferation over 24 hours in surviving RECs (Fig. 6a; IR vs. Control *P<0.05). The proliferative capacity was reduced to control levels in KZ-41 treated irradiated RECs (IR+KZ-41 vs. IR, **P<0.05). In A549 cells (human alveolar type II-like lung epithelial cell line), radiation-induced migration and proliferation are associated with up-regulated p38MAPK phosphorylation, paxillin-dependent cytoskeletal changes and nascent focal adhesion localization [57]. In fact, irradiation has been shown to specifically induce both the expression and phosphorylation of paxillin in A549 cells [58]. Radiation-induced REC proliferation directly correlated with a significant induction in tyrosine phosphorylation of paxillin (Y118) 24 hours following IR (Fig. 6b; *P<0.05). Irradiated RECs treated with either KZ-41 or the p38MAPK inhibitor SB202190 had significantly reduced radiation-induced phosphorylation of paxillin (Fig. 6b; #P<0.05). Our data suggest that REC proliferative capacity and cellular motility following radiation exposure are linked to p38MAPK-dependent paxillin phosphorylation and that KZ-41 lessens radiation-induced REC proliferation via p38MAPK inhibition.

### KZ-41 reduces avascular area and pathological neovascularization in the murine OIR model

**Figure 7:**
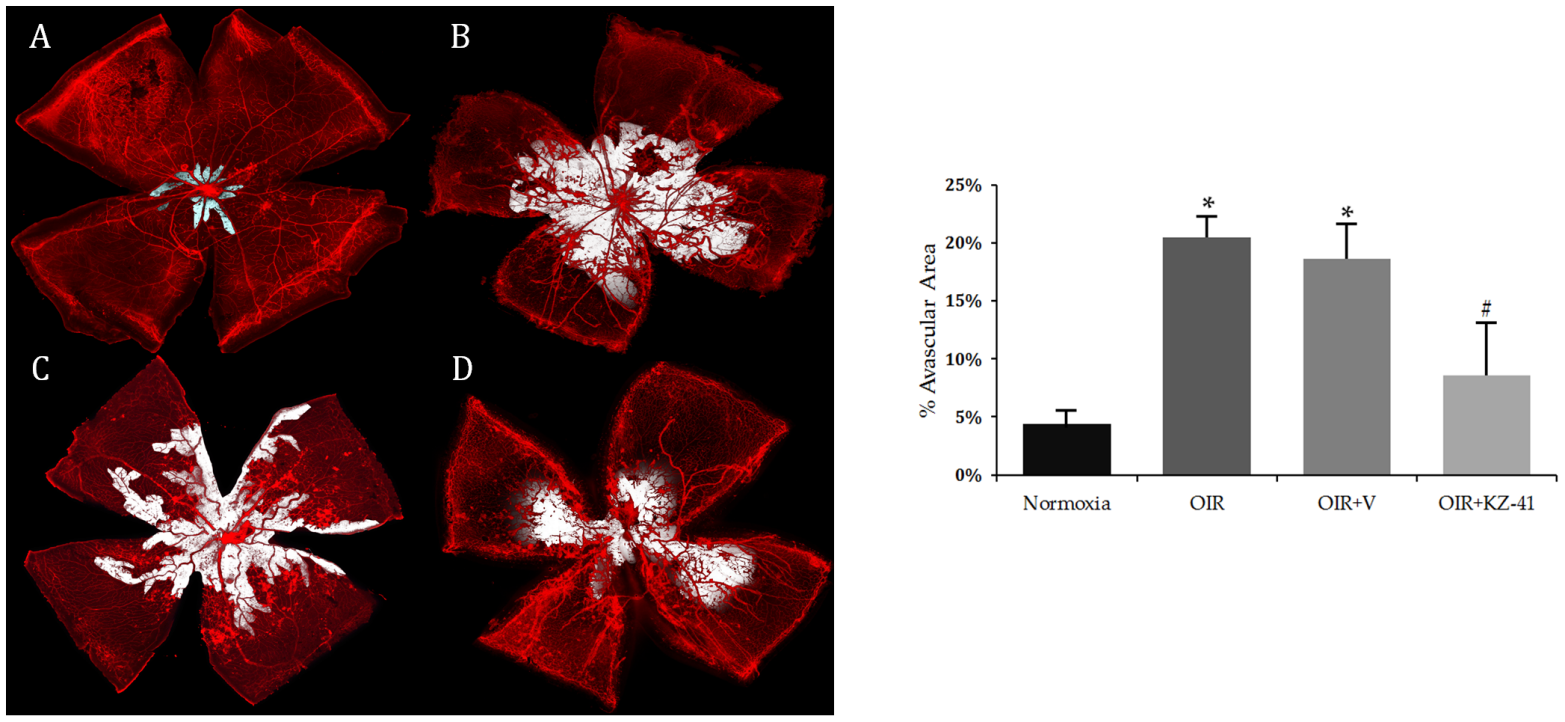
KZ-41 reduces ischemic retinopathy/RNV: Avascular area. A–D) representative flat-mounted retinas stained for endothelial cells using isolectin-B4 (red) from eyes harvested at P17: Normoxia, OIR, OIR+V, OIR+KZ-41, respectively. Mice received daily ocular administration of either KZ-41 (100 mg/kg) or vehicle (ocular nanoemulsion) from P12 to P17. Avascular area was determined using software-assisted analysis; shown in white. OIR mice show significant avascular area as compared to normoxia controls (*P<0.001). KZ-41 lowered area percent avascular area by nearly 50% (#P<0.001). Images were acquired at 10x magnification and digitally stitched together to show the entire retinal vasculature. Data represent mean (± SD). N = 5/group.

**Figure 8:**
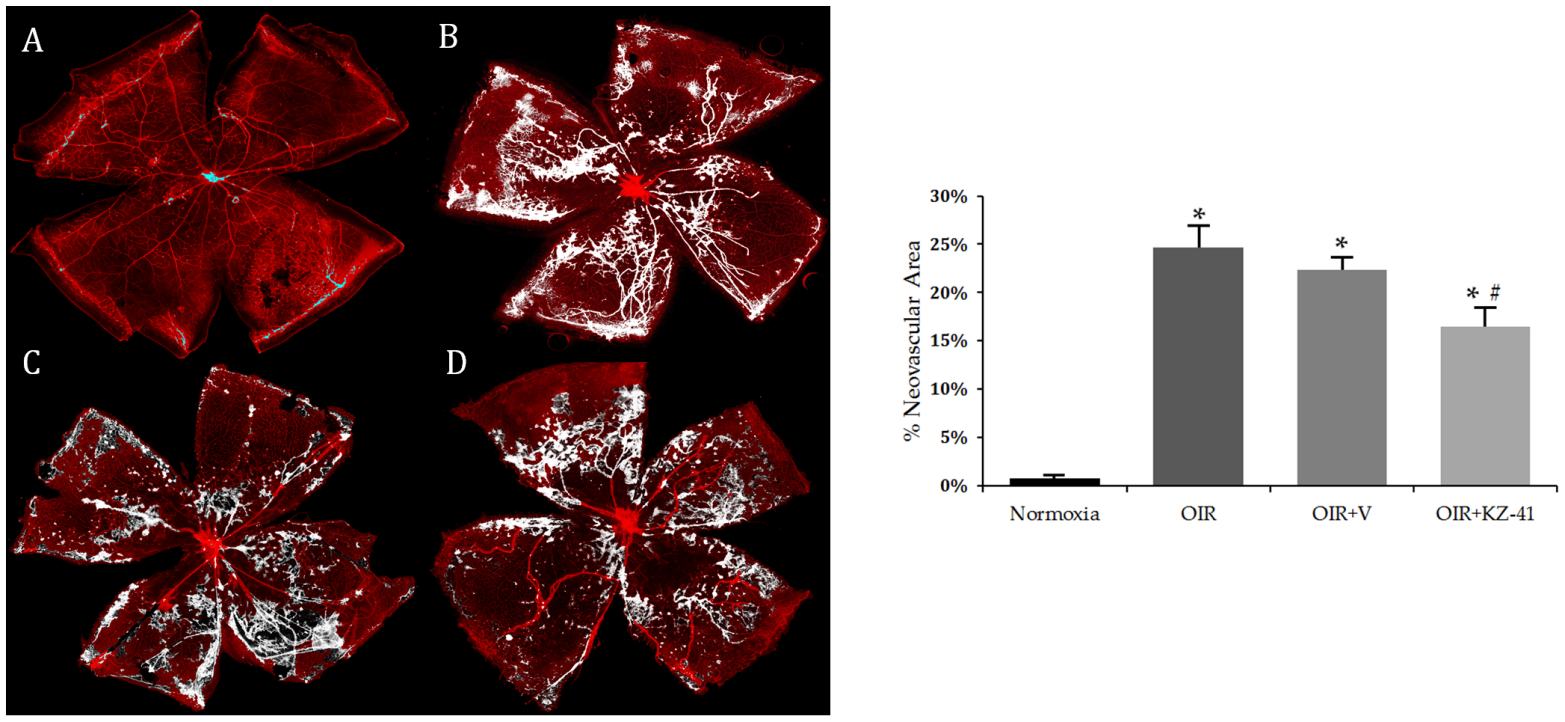
KZ-41 reduces ischemic retinopathy/RNV: Neovascular area. A–D) representative flat-mounted images of P17 retinas stained for endothelial cells using isolectin-B4 (red) were analyzed for neovascular tuft formations: Normoxia, OIR, OIR+V, OIR+KZ-41, respectively. Mice received daily ocular administration of either KZ-41 (100 mg/kg) or vehicle (ocular nanoemulsion) from P12 to P17. Analysis was performed after setting threshold limits to disregard non-neovascular networks and larger vessels in and around the optic disc (high intensity neovascular tufts shown in white). Both OIR groups (untreated and vehicle-treated) showed extensive tufting compared to normoxia controls (*P<0.005). KZ-41 lowered area percent neovascular tufts by ∼30% from OIR+Vehicle (#P<0.01). Images were acquired at 10x magnification and digitally stitched together to show the entire retinal vasculature. Data represent mean (± SD). N = 5/group.

The most frequently used *in vivo* model for studying the effect of genomic or pharmacologic manipulation of key signaling proteins on the natural history of proliferative retinopathies (*e.g.*, RR) is the murine oxygen-induced retinopathy (OIR) model [40], [41], [43]. In the murine OIR model, retinal expression of phosphorylated p38MAPK is enhanced [59]. We used the OIR model to test the hypothesis that KZ-41 would prevent RNV driven by oxidative stress and ischemic injury. Mouse pups received daily ocular administration of either KZ-41 (100 mg/kg; treated eye) or vehicle (ophthalmic NE; contralateral eye) from P12 to P17. As shown in Figure 7 (A–D; Normoxia-N17, OIR17-*untreated*, OIR17+*Vehicle*, and OIR17+*KZ-41*), hyperoxia led to significant vaso-obliteration of the central retina of mouse pups (P7–P12). Both untreated and vehicle-treated retinas showed significantly larger avascular areas surrounding the optic disc as compared to normoxia controls (Fig. 7A–C, 20.5±1.8, 18.6±3.1 vs. 4.4±1.1 AV% area, *P<0.001). Significant neovascularization was also noted in both untreated and vehicle-treated retinas when compared to normoxia controls (Fig. 8A–C, 24.7±2.3, 22.3±1.4 vs. 0.76±0.28 NV% area, *P<0.005). Neither total avascularity nor neovascularization differed between untreated and vehicle-treated retinas (Figs. 7B,C and 8B,C; P>0.05). KZ-41 significantly reduced avascularity (8.6 vs. 18.6 AV% area, #P<0.001) and neovascularization (16.5 vs. 22.3 NV% area, #P<0.01) compared to vehicle-control retinas (Figs. 7C,D and 8C,D). These results suggest that KZ-41 reduces the extent of pathological RNV while not affecting normal revascularization under OIR conditions.

## Discussion

Severe microvascular injury has been touted as the primary mechanism in the pathogenesis of radiation-induced tissue damage [46]. Studies have emphasized the primary mechanism of retinal injury involves leukocyte entrapment and accumulation within microvascular circulation with resultant capillary closure and subsequent ischemia [45]. ICAM-1 is one of the most recognizable initiators of leukocyte-endothelial cell adhesion [50] and has been highly correlated with ocular inflammatory disorders such as diabetic retinopathy, retinopathy of prematurity, and RR) [12]–[14], [60], [61]. ICAM-1 not only facilitates the adhesion and transmigration of circulating leukocytes into the site of tissue damage but also exacerbates inflammation through the same signaling cascades driving its surface expression, p38MAPK.

In our present study, we have demonstrated that absorbed doses of radiation to the human REC provokes an inflammatory response characterized by rapid induction in p38MAPK stress kinase-mediated pathways and downstream effectors, *e.g.*, tumor suppressor, p53 and ICAM-1. We have previously demonstrated that the quinic acid derivative KZ-41 modulates cellular responses to genotoxic stress via mechanisms involving disruption of p38MAPK signal transduction [16], [24], [62]. In the current study, we extend these findings by demonstrating that KZ-41 also modulates p38MAPK activity in the irradiated REC (Fig. 4). KZ-41 blunts p38MAPK activity following radiation exposure, reducing p53 activation, ICAM-1 expression, and adhesion of leukocytes to the inflamed RECs.

Our results show an ICAM-1 dependent increase in leukocyte adhesion in radiation-induced injury to RECs. Disruption of ICAM-1 signaling, either through gene knockout or antibody blockade, prevents VEGF-mediated pathological angiogenesis [63], [64]. Irradiation-induced activation of p38MAPK is not limited to promoting acute inflammatory responses (*i.e.*, leukocyte adhesion), but is also capable of propagating chronic inflammatory phenotypes, such as the migration and proliferation of cells through cytoskeletal protein effectors such as focal adhesion kinases (FAK) and scaffolding protein, paxillin [58].

Focal adhesions (FAs) containing paxillin•FAK complexes coordinate traction and retraction of cellular protrusions and direct movement of dividing and proliferating cell populations [65]. Disruptions in paxillin-coordinated cellular movement through site-directed mutagenesis of key paxillin phosphorylation sites Y31/Y118 or serine 178 reduces FA turnover kinetics and hinders the proliferative/migratory phenotype of endothelial cells [65], [66]. Additionally, genetic knockdown of focal adhesion proteins prevents nascent focal adhesion formation thereby reducing VEGF-mediated RNV in the murine OIR model [67]. Non-specific p38MAPK inhibition has also been shown to reduce vaso-obliteration and neovascular tuft formation in OIR [68]. Not surprisingly, both p38MAPK and paxillin are key regulators of the VEGF-dependent angiogenic response in endothelial cells [69], [70]. We have shown that in RECs, radiation injury triggers proliferative cell motility through p38MAPK-dependent activation of paxillin and the treatment of KZ-41 prevents this proliferative phenotype by reducing p38MAPK-dependent paxillin phosphorylation (Fig. 6). Uncoupling p38MAPK and paxillin signal transduction thus offers a potential strategy to design novel therapeutics for the treatment of pathologic retinal neovascularization.

The aforementioned data, together with our own, suggest a potential causal link between p38MAPK and proliferative retinopathy following radiation injury. We hypothesized that KZ-41 would halt progression of pathologic neovascularization in the murine OIR model. We tested this hypothesis by first developing an ocular nanoemulsion drug delivery system to facilitate non-invasive multiple drug dosing in the neonatal mouse. The ocular nanoemulsion appears not to affect primary study endpoints since both untreated and vehicle-treated eyes of OIR mice demonstrated expansive areas of the retina devoid of vascularity around the optic disc as well as extensive neovascular tufting, all hallmark signs of retinopathy (Figs. 7 and 8). In contrast, daily ocular administration of KZ-41-loaded nanoemulsion during this period of hypoxia decreased ischemic retinopathy near the optic disc and significantly reduced neovascular tufting. Moreover, the effect of KZ-41 appears to be local, as opposed to systemic, since the KZ-41-treated eyes had significantly less pathologic RNV features compared to the contralateral eye, which received vehicle alone.

**Figure 9:**
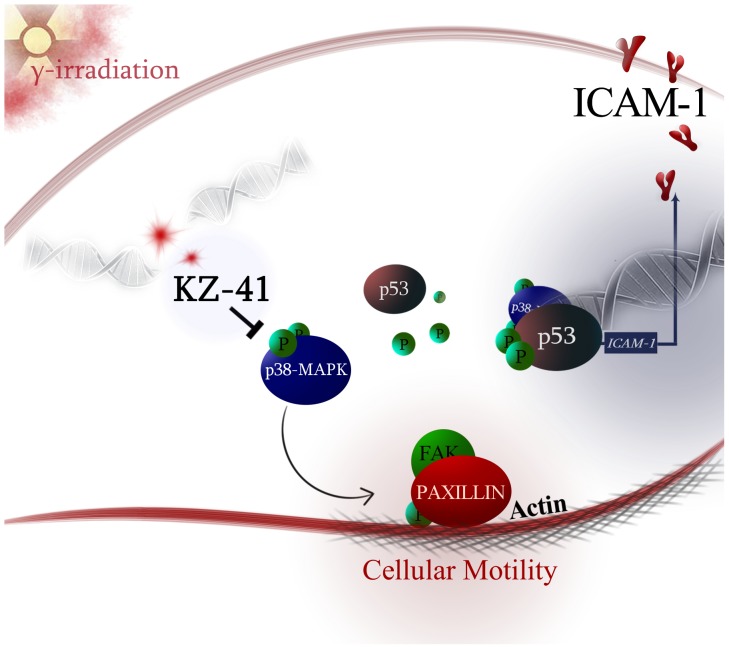
Model of KZ-41 radioprotective mechanism-of-action. Gamma-(γ) radiation-induced DNA double strand breaks (DSBs) trigger phosphorylation of p38MAPK which in turn results in p53 accumulation enhances ICAM-1 surface levels and incites a proliferative/migratory phenotype through paxillin phosphorylation. KZ-41 reduces phospho-p38MAPK and effectively uncouples p38 MAPK signaling to reduce REC inflammation and halt aberrant cellular motility. Therefore, KZ-41 is able to protect RECs against acute radiation injury and the resultant dysfunction of the retinal vasculature.

In conclusion, the human REC radiation injury response includes activation of p38MAPK-dependent signal transduction pathways that culminate in a pro-angiogenic phenotype. Coordinated cytoskeletal rearrangements required for migration and proliferation are supported by enhanced expression and activation of cell adhesion molecules and scaffold proteins responsible for recruiting other proteins to focal adhesions. Further, our data suggest that KZ-41, through specific modulation of p38MAPK and its downstream effectors (*i.e.*, p53, ICAM-1 and focal adhesion protein, paxillin), effectively reduces both the inflammation associated with radiation and the pathological manifestations of p38MAPK-dependent RNV (Fig. 9). Additional insight into the mechanism(s) of action, including identification of the cellular target, are required to fully explore the therapeutic potential of quinic acid derivatives such as KZ-41 in treating retinal inflammatory disorders.
